# Feasibility and efficacy of active breathing coordinator assisted deep inspiration breath hold technique for treatment of locally advanced breast cancer

**DOI:** 10.1002/acm2.13893

**Published:** 2022-12-31

**Authors:** Sean All, Bo Zhao, Steven Montalvo, Christian Maxwell, Christopher Johns, Xuejun Gu, Asal Rahimi, Prasanna Alluri, David Parsons, Tsuicheng Chiu, Samuel Schroeder, D. Nathan Kim

**Affiliations:** ^1^ Department of Radiation Oncology University of Texas Southwestern Medical Center Dallas Texas USA; ^2^ Department of Radiation Oncology Memorial Sloan Kettering Cancer Center New York New York USA; ^3^ University of Texas Southwestern Medical School Dallas Texas USA; ^4^ Department of Radiation Oncology Stanford University Palo Alto California USA; ^5^ UnityPoint Health Department of Radiation Oncology John Stoddard Cancer Center Des Moines Iowa USA

**Keywords:** ABC, DIBH, Locally Advanced Breast Cancer, 3‐field technique, field matching

## Abstract

**Background:**

Active breathing coordinator (ABC)‐assisted deep inspiration breath hold (DIBH) is an important organ sparing radiation therapy (RT) technique for left‐sided breast cancer patients. Patients with advanced breast cancer undergoing chest wall and regional nodal irradiation often require a field matching technique. While field matching has been demonstrated to be safe and effective in free breathing patients, its safety and accuracy in DIBH/ABC use has not been previously reported.

**Purpose:**

To report the accuracy, feasibility, and safety of field matching with ABC/DIBH for patients receiving breast/chest wall irradiation with nodal irradiation using a three‐field technique.

**Methods:**

From December 2012 to May 2018, breast cancer patients undergoing ABC/DIBH‐based RT at a single institution were reviewed. For each fraction, the amount of overlap/gap between the supraclavicular and the tangential field were measured and recorded. Patient characteristics, including acute and delayed skin toxicities, were analyzed.

**Results:**

A total of 202 patients utilized ABC/DIBH and 4973 fractions had gap/overlap measurements available for analysis. The average gap/overlap measured at junction was 0.28 mm ± 0.99 mm. A total of 72% of fractions had no measurable gap/overlap (0 mm), while 5.6% had an overlap and 22.7% a gap. There was no significant trend for worsening or improvement of gap/overlap measurements with increasing fraction number per patient. OSLD measurements were compared to the planned dose. The median dose 1 cm above the junction was 106% ± 7% of planned dose (range 94%–116%). One centimeter below the junction, the median dose was 114% ± 11% of planned dose (range 95%–131%). At the junction, the median dose was 106% ± 16.3% of planned dose (range 86%–131%). Acute skin toxicity was similar to historically reported values (grade 3, 5.4%, grade 4, 0%).

**Conclusion:**

ABC‐assisted DIBH is a safe and technically feasible method of delivering RT in the setting of complex matching field technique for breast and regional nodal treatments.

## 1 INTRODUCTION

Patients undergoing radiation for locally advanced breast cancer often require treatment fields that encompass both the breast/chestwall and regional nodal areas. Due to this, a field matching technique is often employed, with each field being irradiated one at a time. This technique has a potential risk of fields overlapping resulting in an overdose or, if not accurately matching, under dosing at or near the match line. In free breathing patients, this matching technique has been demonstrated to be safe and effective.^1^ Deep inspirational breath hold (DIBH) is an important technique that is utilized during radiation therapy to facilitate cardiac sparing. During DIBH, the patient takes inspiration to a particular threshold to expand the lungs, causing the heart to pull away from the chest wall and thereby lowering its radiation exposure.^2^, ^3^ In patients with left‐sided breast cancer, DIBH significantly reduces mean cardiac dose and, thus, is a standard technique for cardiac‐sparing.^4^ While there are several methods of performing DIBH during radiation therapy, active breathing coordinator (ABC) technology has been employed at our institution to ensure reproducibility of breath holds. ABC incorporates a spirometer linked to a computer, which allows for real time monitoring of the breathing cycle and ensures that the radiation delivery is only active when the patient has met the appropriate inspiratory threshold.^2^, ^3^ This is accomplished by setting a threshold that is unique to each patient's tolerance, and once set, allows the device to control the amount of air in each breath to create a reproducible action. While explored in other breath hold techniques, the safety of employing field matching technique with ABC/DIBH remains poorly studied.^5^

Given the potential risk of overdosing or under dosing in these patients at the field junction, an important consideration is radiation‐induced toxicity of the skin, as it is influenced by dose received and duration of radiation exposure.^6^ Typically, patients can experience acute dermatitis reactions in the treatment field, primarily grade 2 or lower by the RTOG skin toxicity scale.^6^ Delayed skin reactions can include fibrosis and, in severe instances, skin ulcerations.^7^ Furthermore, if significant overlap occurred at the match line, a high‐grade acute dermatitis during radiation therapy may occur as well as increased risks of developing late brachial plexopathy. Therefore, manifestations of acute and delayed skin toxicities can be used as a surrogate measure of the degree of overlap of radiation fields at the match line. The purpose of this study is to determine the safety of ABC/DIBH for locally advanced breast cancer patients requiring multi‐field matching techniques and to provide data on its accuracy and reproducibility.

## 2 METHODS

The Institutional Review Board (IRB) approved registry protocol (STU 052012‐019) at our institution, permitted this study. From December 2012 to May 2018, all patients who underwent ABC/DIBH at our institution were evaluated. Clinicopathologic variables such as age, sex, body mass index (BMI), tumor stage, treatment dates, radiation treatment dose, local recurrence date, regional recurrence date, and distant recurrence date were extracted retrospectively from the electronic medical record (EMR) system.

### 2.1 Patient simulation technique and treatment planning

All patients were simulated using ABC/DIBH (Elekta, Stockholm, Sweden) as a cardiac tissue sparing technique. Prior to computed tomography (CT) simulation, radiation therapists provided patients with a brief tutorial that included determining the patient's unique inspiratory threshold level. The ABC threshold level was customized to accommodate the patient tolerance that enabled performance of DIBH comfortably without fatigue. Two CT image sets were acquired during simulation: one with free breathing and the other with the patient performing ABC/DIBH. The two scans were compared to determine the relative benefit of the breath hold in regards to cardiac sparing. A three‐dimensional technique was used with matching of the inferior border of the supraclavicular (SCV) field to the superior border of the opposed tangential breast/chestwall fields. In some cases, wide tangents were employed to include internal mammary nodes (IMN), where indicated. While there was no classic field feathering performed during treatment planning, a field‐in‐field technique was used with single or two energy photon beams to allow for more dose homogeneity throughout the plans. These individual SCV and tangent plans with field‐in‐field technique typically generated a gradient over the matchline for each plan, which mitigated potential for overdoses within a small range of set up error, which simulated a “feathering” effect. The isocenter was placed at the match line between the inferior border of the SCV field and the superior border of the tangential fields.

### 2.2 Patient setup and matchline measurement

During initial treatment setup, patients performed ABC/DIBH. During the breath hold, the light field was displayed on the patient's skin and the radiation therapist manually drew the match line and field. The match line was drawn at the inferior edge of the SCV light field. This process was repeated throughout the course of treatment to maintain the demarcation of the match line and prevent diminished markings. The SCV field was typically treated first. Upon completion of treating the first field, therapists would then load the medial tangent field and project the light field of the medial beam onto the patient. The patient was then asked to perform another DIBH. If the superior light field border of the tangent field did not align with the marked match line, the couch positioning was adjusted longitudinally to manually match this line. The workflow decision to treat the SCV field first and then shift the patient longitudinally to match the light field before treating with the tangent fields was done in an effort to account for any patient motion between the SCV field treatment and the tangent field treatment. Each patient went through a “verification” set up session prior to delivery of first treatment, where the isocenter from the free‐breathing set up and the DIBH treatment matchline were marked on the patient's skin. The treatment set‐up was mono‐isocentric, and therefore the matchline coincided with the inferior border of the SCV field, and the superior border of the tangential fields respectively. Therefore, on each treatment day, for each field, the patient was set up on isocenter, and then on DIBH, the light field was monitored to check for match to the previously set matchline. Therapists prospectively recorded the degree of couch adjustment as an indicator of gap or overlap. A positive junction measurement value indicated a gap and a negative junction measurement value indicated a field overlap.

### 2.3 Absolute dose measurement at matchline

**FIGURE 1:**
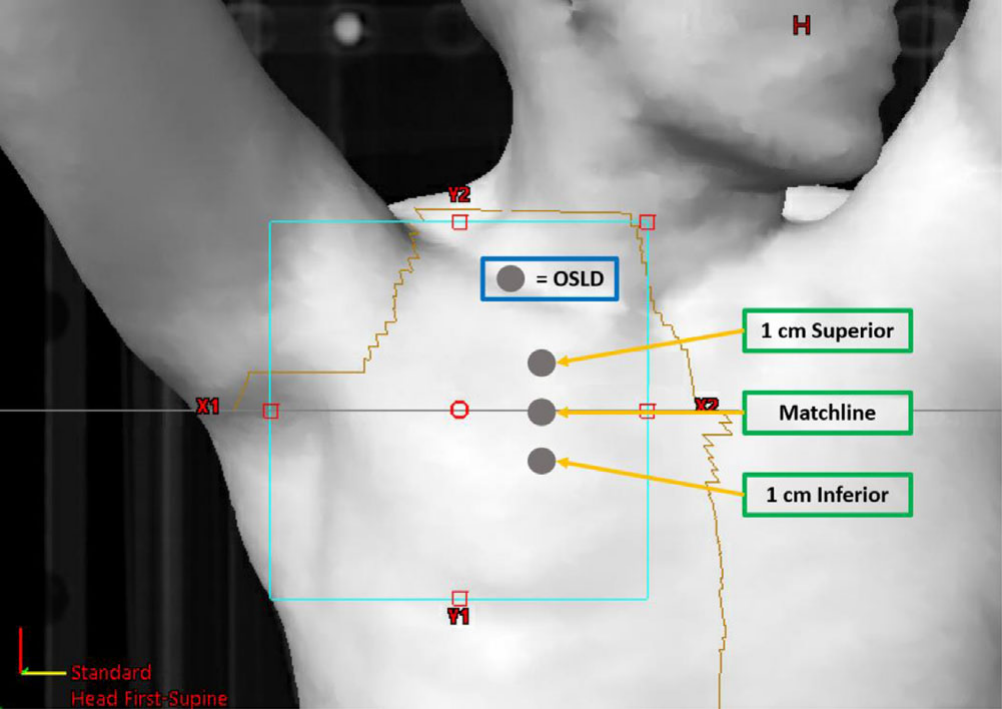
Diagram depicting standardized locations for OSLD dose measurements

Optically stimulated luminescence dosimeters (OSLD; Landauer, IL) were used on 10 patients treated with matched fields to measure in vivo dosimetry to verify the match line dose in addition to matchline gap measurements. Three OSLDs were placed along the superior/inferior direction crossing isocenter: 1 cm superior to the match line, at the match line, and 1 cm inferior to the match line (Figure 1). These measurements were repeated three times throughout the treatment course. The measurement was compared with the calculated dose from treatment planning system (TPS; Eclipse, Varian, CA). The TPS dose was estimated at 5 mm depth below the skin at the locations where the OSLDs were placed. The expected dose was modeled in Eclipse as a point dose at 5 mm depth at isocenter (and 1 cm above and below) on the summed plan.

### 2.4 Toxicity

Acute skin toxicities were prospectively recorded by treating radiation oncologists utilizing the common terminology criteria for adverse events (CTCAE) version 4.0 acute toxicity scale incorporated into the EMR as part of weekly on‐treatment visits. Delayed skin toxicities were extracted by chart review from patient's EMR of visits occurring at least 3 months after treatment completion.

## 3 RESULTS

A total of 202 patients with breast cancer were treated at our institution with ABC/DIBH using three field technique and were included in this study. Patient characteristics are detailed in Table 1. While the primary intent of ABC technology is intended to facilitate cardiac sparing in left‐sided breast cancer patients, we occasionally employ it for patients with right‐sided disease due to anatomic challenges or for women with bilateral disease. In our cohort, 192 patients had left‐sided disease, 6 had right‐sided disease, and 4 had bilateral disease (Table 1). More than half of the patients (69%) received neoadjuvant chemotherapy.

**TABLE 1:** Patient and treatment characteristics

**Characteristic**	
BMI (Mean, SD)	29.8 ± 6.0
**Age (Mean, SD)**	51.0 ± 10.4
Less than or equal to 40	39 (19.3%)
41–50	55 (27.2%)
51–60	67 (33.2%)
61–70	35 (17.3%)
Greater than or equal to 71	6 (3.0%)
**Laterality (*n*, %)**	
Left	192 (95.0%)
Right	6 (3.0%)
Bilateral	4 (2.0%)
**Histology (*n*, %)**	
Infiltrating Ductal	174 (86.1%)
Infiltrating Lobular	16 (7.9%)
Inflammatory	7 (3.5%)
Neuroendocrine	1 (0.5%)
Multiple	4 (2.0%)
**Clinical Staging (*n*, %)**	
T1	20 (9.9%)
T2	61 (30.2%)
T3	67 (33.2%)
T4	20 (9.9%)
N0	54 (26.7%)
N1	101 (50.0%)
N2	11 (5.4%)
N3	15 (7.4%)
M0	164 (81.2%)
M1	4 (1.5%)
**Neoadjuvant Chemotherapy (*n*, %)**	
Yes	139 (68.8%)
No	64 (31.7%)

**FIGURE 2:**
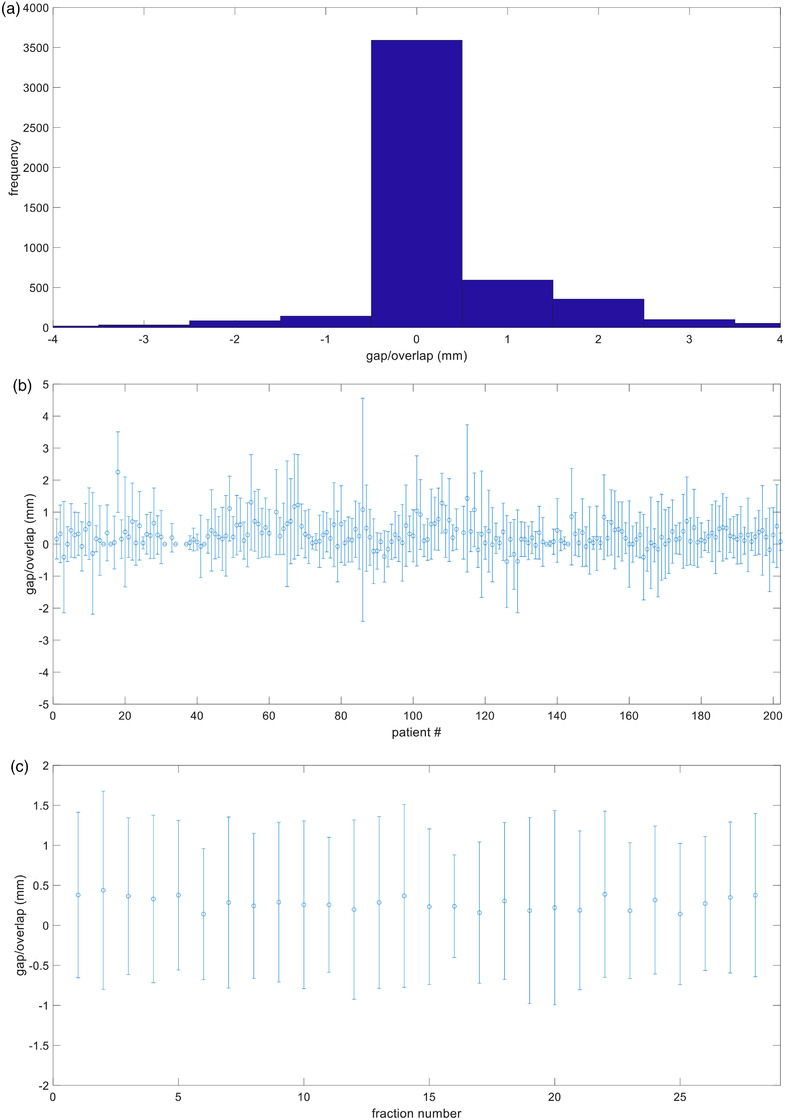
(a) Histogram of the gap measurement. Positive value indicates gap and negative value indicates overlap. (b) Measurement statistic of each patient. (c) Mean ± Standard Deviation of Overlap/Gap match line measurements at each fraction. This graph shows how the overlap/gap measurement changed as a function of time

A total of 4973 junction measurements were recorded. The average gap at match line was 0.28 ± 0.99 mm (median = 0; range –6.0 to 7.0 mm). Figure 2a depicts a histogram distribution of the junction measurements. Most fractions (71.7%) had no measurable gap or overlap between fields and more fractions had gaps (22.7%) than overlap (5.6 %). Figure 2b depicts the variation in measurements between each individual patient. Between fractions, there was minimal variability in the amount of gap or overlap seen (Figure 2c) indicating that the accuracy of match line set up with ABC/DIBH remained consistently reproducible throughout the entire course of radiation treatment. Although the overwhelming majority of patients were treated with couch adjustment to eliminate the gap or overlap, 11 patients that had gap/overlap did not undergo couch adjustment prior to treatment.

**FIGURE 3:**
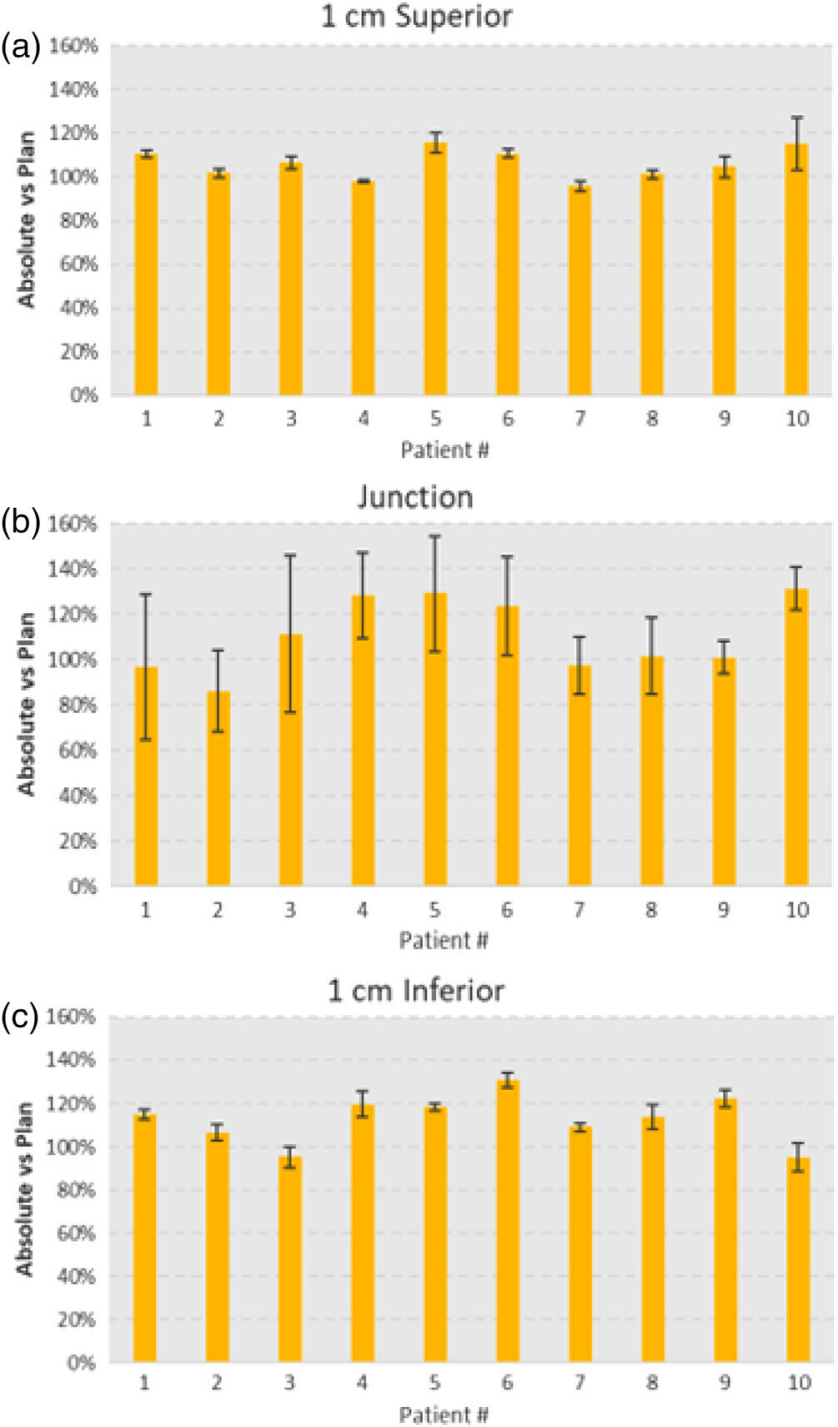
Comparison of OSLD measurements with Eclipse treatment planning system dose predictions in ten patients treated with ABC/DIBH technique. (a) Measurements taken at 1 cm superior to, (b): at, and (c): 1 cm inferior to the junction. Error bars show the standard deviations of three measurements taken at each location

We correlated TPS‐calculated surface dosimetry measurements with OSLDs (Figure 3a–c). Table 2 depicts the average OSLD doses (as a percentage of total dose) for each measurement point. The measured dose at the match line was 104.6% ± 34.8%. The dose measurements were more consistent and closer to the TPS calculated values at the superior location (1 cm superior: 99.6% ± 26.1%) but slightly more variable at the inferior location (1 cm inferior: 105.7% ± 29.1%).

**TABLE 2:** OSLD measured dose relative to predicted dose at specified measurement locations in 10 unique patients

**Dose (%)**	**1 cm Superior**	**Matchline**	**1 cm Inferior**
**Average**	99.6% ± 26.1%	104.6% ± 34.8%	105.7% ± 29.1%
**Median**	106% ± 7%	106% ± 16.3%	114% ± 11%
**Min**	94%	86%	95%
**Max**	116%	131%	131%

There were no grade 4 toxicities in this cohort and grade 3 toxicity was only seen in eleven patients (5.4%) patients. Most patients (94%) experienced ≤ grade 2 dermatitis (Table 3). No patients experienced long‐term late high grade (≥ grade 3 and above) skin toxicity. No incidence of significant match line dermatitis or fibrosis was found on retrospective review of toxicity data.

**TABLE 3:** Acute radiation dermatitis (RTOG Skin Toxicity) in patients treated with ABC/DIBH technique

Grade	Occurrence
0	9 (4.4%)
1	55 (27.1%)
2	127 (62.6%)
3	11 (5.4%)
4	0

## 4 DISCUSSION

DIBH has long been known to reduce cardiac dose in left‐sided breast cancer patients receiving radiotherapy.^2^, ^8^ We have previously shown that utilization of ABC/DIBH in patients with unfavorable cardiac anatomy who required IMN nodal coverage resulted in reduced mean heart dose.^4^ However, whether such gains in cardiac sparing are associated with inconsistent field matching (which could result in significant cold/hot spots and/or increased skin toxicity) remains poorly studied. This study provides evidence that ABD/DIBH is safe when complex field matching is necessary in radiation of the breast or chest wall. Furthermore, most fractions had incalculable junction measurements, indicating high accuracy and reproducibility of patients’ DIBHs. Most patients had minimal interfractional and intrafractional variability. Inconsistent breath holds contributed to infrequent instances of intrafractional variability and are likely related to multiple factors. Anecdotally, language barriers and patient fatigue are two common contributors to inconsistencies in breath‐hold, although we did not assess this formally in this study. Additionally, patient visual feedback has also been shown to improve the quality of breath holds.

We sought to describe the measured surface dosimetry for patients undergoing ABC/DIBH and found acceptable deviations from prescription dose. Our findings indicate minimal dose variability 1 cm from junctional match line but a 34.8% variability at the junctional match line. These findings are in agreement with prior studies, which compared free breathing to voluntary DIBH technique in patients receiving tangential breast/chest wall radiation and regional nodal radiation using in vivo dosimetry, where junction matchline variability was 30.5%.^1^ Notably the error bars of the dose measurements at match line were higher compared to those at 1 cm superior and inferior to the match line. These variations in the measured junction dose were found to be statistically significant for nearly half of the patients at the superior (4/10 patients) and inferior (6/10 patients) measurement points after an F‐test was performed for each of the 10 patients. Inconsistent DIBHs not only between fractions but also intrafractionally are both likely contributors to this observed variability. Additionally, the matchline junction measurements have a high dose gradient and accurate measurement is dependent on precise dosimeter placement. Thus, even small shifts in OSLD placement may lead to large variations in dose readings. Furthermore, the accuracy of the specific OSLDs used in this analysis is ±10% per the manufacturer report. Finally, the numerical variability in dose measurements observed in our study was not clinically significant. In addition, the rates of acute and late skin toxicity in our patients were similar to historic controls. This may be secondary to the very low rates of overlap and the majority of patients having no measurable gap or overlap as measured clinically.

Our study showed excellent reproducibility. The average gap measured at match line was submillimeter despite contributions from daily setup changes and measurement errors. How errors in setup can be further optimized remains an active area of investigation. Some investigators have integrated infrared optical tracking with a spirometry device to develop 3‐D coordinates of different trackers on patients to monitor DIBH consistency with modified tracking structures with sub‐5 mm accuracy.^9^, ^10^ Another method incorporated cinematographic mode of an electronic portal‐imaging device to monitor voluntary DIBH quality by capturing images during treatment delivery and measuring beam distance to chest wall.^11^ Other strategies to assist with reproducibility of DIBH include, providing supplemental oxygen, which improved breath hold performance and reduced participant fatigue, minimizing the number of breath holds required in a treatment and an overall increase in patient comfort.^12^

## 5 CONCLUSION

ABC/DIBH is an accurate, safe, technically feasible and reproducible method for delivering radiation in the setting of complex matching field technique for breast/chest wall and regional nodal treatments and is not associated with increases in acute or late skin toxicity. These results provide a foundation for integrating this technology into clinical practice.

## AUTHOR CONTRIBUTIONS

Bo Zhao, Christian Maxwell, Xuejun Gu, Tsuicheng Chiu, and Nathan Kim helped in conceptualization and project administration; Bo Zhao, Tsuicheng Chiu, D. Nathan Kim, Asal Rahimi, Prasanna Alluri, David Parsons, Xuejun Gu, and Christian Maxwell contributed to data curation and analysis; Sean All, Steven Montalvo, Samuel Schroeder, Bo Zhao, Christian Maxwell and D. Nathan Kim wrote the main manuscript text, assisted with additional data analysis and interpretation, and preparation of the figures. All authors read, and approved the final manuscript.

## CONFLICT OF INTEREST

No conflicts of interest.
